# Neurobiological correlates of violence perception in martial artists

**DOI:** 10.1002/brb3.1276

**Published:** 2019-03-24

**Authors:** Maria Schöne, Stephanie Seidenbecher, Leonardo Tozzi, Jörn Kaufmann, Hendrik Griep, Daniela Fenker, Thomas Frodl, Bernhard Bogerts, Kolja Schiltz

**Affiliations:** ^1^ Department of Psychiatry and Psychotherapy Otto‐von‐Guericke University Magdeburg Germany; ^2^ Salus‐Institute Magdeburg Germany; ^3^ Department of Psychiatry and Institute of Neuroscience Dublin Ireland; ^4^ Department of Psychiatry and Behavioral Science Stanford University Stanford California; ^5^ Department of Neurology Otto‐von‐Guericke University Magdeburg Germany; ^6^ Center for Behavioral Brain Sciences Otto‐von‐Guericke University Magdeburg Germany; ^7^ German Center for Neurodegenerative Diseases Magdeburg Germany; ^8^ Department of Forensic Psychiatry Psychiatric Hospital of the University of Munich Munich Germany

**Keywords:** aggression, functional magnetic resonance imaging, martial arts, violence

## Abstract

**Objectives:**

The direct exertion as well as the visual perception of violence can have a hedonistic effect and elicit positive arousal in predisposed individuals. This appetitive aspect of aggression in healthy subjects has been neglected in psychiatric research so far.

**Methods:**

Using functional magnetic resonance imaging, we tested whether subjects trained in sports with a violent component (martial arts) show altered brain responses in reward‐associated brain areas when compared to controls. Sixteen martial artists (e.g., boxing, mixed martial arts) and 24 controls watched violent versus neutral pictures while performing a cognitive cover task. Subjects’ aggressiveness was assessed by the aggressiveness factors questionnaire (FAF).

**Results:**

While watching violent pictures, martial artists had a stronger activation in the left amygdala than controls. Within the martial artist group however, there was an inverse correlation between activation in the left amygdala and degree of aggressiveness.

**Conclusions:**

Higher amygdala activation while watching violent pictures might reflect that perception of violence conveys increased salience to martial artists as compared to controls. The inverse correlation between amygdala activation and aggressiveness within the martial artist group might be explained by the assumption that the more aggressive martial artists may be more accustomed to violent situations leading to a down‐modulation of amygdala activation. Appetitive aggression should be taken into account as a factor contributing to violence.

## 1 INTRODUCTION

### 1.1 The general aggression model

The general aggression model (GAM) is a comprehensive framework to understand the origin of aggression and describes the impact of social, cognitive, developmental, personality, and biological factors (Allen, Anderson, & Bushman, 2018). The GAM consists of distal and proximate causes. Distal processes include biological (e.g., testosterone level) and environmental modifiers (e.g., an antisocial peer group) influencing personality that, in turn, influences proximate processes. Proximate processes include relatively stable person factors (e.g., high trait anger), situational factors (e.g., provocation), the present internal state of the person (i.e., affect, cognition, and arousal) as well as appraisal and decision processes to act aggressively or not. In addition to the assumptions of the GAM, aggression can also incorporate a hedonistic (appetitive) component (e.g., Chester & DeWall, 2016). The appetitive component of aggression was not taken into account in the GAM so far.

### 1.2 Appetitive aggression and its distinction from other forms of aggression

Elbert and his research group (e.g., Elbert, Weierstall, & Schauer, 2010) describe appetitive aggression as hedonistically motivated and argue that predisposed individuals behave violently because they experience the violent act itself as fascinating, exciting, or even euphoric. Retaliation/revenge (Beyer, Münte, Göttlich, & Krämer, 2015; Buades‐Rotger, Brunnlieb, Münte, Heldmann, & Krämer, 2016), “Schadenfreude” caused by an envied person's misfortune (Takahashi et al., 2009), or pleasure by inducing pain in a provocative person (Chester & DeWall, 2016) do not exactly represent appetitive aggression because appetitive aggression implies an intrinsic motivation to act violently (Köbach, Schaal, & Elbert, 2015). Reward‐associated aggression may be a universal trait detectable in all people (Moran, Weierstall, & Elbert, 2014). Predisposed individuals (e.g., hooligans or martial artists like boxers or wrestlers) enjoy acting violently because of the reward effect of this behavior itself (Köbach et al., 2015) and might search for situations in which they can act violently. Not only the direct exertion but also the perception of violence frequently has an appetitive aspect. For example, many people watching violence in the media appear to be fascinated (Elbert et al., 2010).

Physical aggression belongs to the natural behavioral repertoire of almost all mammalians (Blair, 2016; Gomez, Verdu, Gonzales‐Megias, & Mendez, 2016). From an evolutionary point of view, it is plausible that animals and humans can exert violence under certain conditions with an appetitive component. Killing of weaker conspecifics improves the perpetrators reproduction rates (Nell, 2006). Therefore, the genes of the successful violent individual will more likely be passed on in contrast to the genes of the victim. Enjoying the act of killing increases the likelihood of this behavior and may have an evolutionary advantage by transmission of genes predisposing to appetitive aggression.

The so far common classification of aggression distinguishes between reactive/impulsive (defensive rage) and proactive/instrumental (predatory attack) aggression (Siegel, Bhatt, Bhatt, & Zalcman, 2007). Individuals engage in reactive aggression to protect themselves against a real or assumed threat. People engage in proactive aggression to intentionally reach a specific goal, for example, to dominate their victim or to achieve a material gain. Both forms of aggressiveness can co‐exist (Rosell & Siever, 2015). An aggressive act may include reactive and proactive as well as appetitive elements (Elbert et al., 2010).

### 1.3 Appetitive aggression in animals

Investigations in animals provide indications for a relationship between physical aggression and dopaminergic activation in the reward system. When a foreign male mouse is put into the cage of another mouse, the intruder is attacked physically by the other mouse (Couppis & Kennedy, 2008; May & Kennedy, 2009). This aggressive behavior goes along with dopamine release in the nucleus accumbens reflecting a reward effect. After injection of a dopamine receptor antagonist, the aggressive behavior disappears (Couppis & Kennedy, 2008). In cats, reactive violence occurs when the medial amygdala is activated, followed by an activation of the medial hypothalamus via the stria terminalis which, in turn, leads to a subsequent activation of the periaqueductal gray in the mesencephalon. Proactive violence is caused by an activation of the lateral amygdala leading to an activation of the lateral hypothalamus via ventral amygdalofugal fibers (Siegel et al., 2007). In rats, hypoactivation in the cortical orbitofrontal cortex, being involved in cognitive control, as well as hyperactivation in the subcortical nucleus accumbens lead to impulsive behavior (Meyer & Bucci, 2016). This cortical‐subcortical imbalance of the reward‐related areas impairs the inhibition of behavior that is rewarding. In fact, this behavior might reflect not only impulsive but also appetitive violence. In primates, the amygdala shows increased activity after delivering the reward (fruit juice) (Bermudez & Schultz, 2010). The activation in the amygdala correlates positively with the reward's amount coding reward magnitude.

### 1.4 Appetitive aggression in humans

So far, appetitive aggression in humans has predominantly been linked to mentally abnormal behavior (Elbert et al., 2010). For example, people with sexually sadistic traits enjoy harming others (Harenski, Thornton, Harenski, Decety, & Kiehl, 2012). Functional deficits in the orbitofrontal cortex of psychopaths have been frequently described (e.g., Anderson & Kiehl, 2014). Further investigations of subjects from the community suggest a hypersensitivity of the reward system as a functional correlate of impulsivity and antisocial behavior; subjects displaying stronger impulsive and antisocial traits show a hypersensitive dopaminergic reaction in the nucleus accumbens during performing a “Monetary incentive delay task” (Buckholtz et al., 2010). Increased activation in the ventral tegmental area when watching violent videos is evident in individuals with stronger interpersonal and affective deficits (Decety, Chen, Harenski, & Kiehl, 2015).

In a previous study (Breitschuh et al., 2018) investigating brain morphology applying structural MRI we found that aggressiveness of martial artists correlated with reduced gray matter in the temporal pole. To our knowledge only one other brain imaging study directly investigated appetitive aggression (Moran et al., 2014), by applying magnetoencephalography. In subjects from the community, delta synchronization in the right parietal‐temporal seems to occur during appetitive but not reactive aggression; activation in this area points toward a better empathic capacity (Decety & Lamm, 2007). The detected relationship between appetitive aggression and less activation in the right parietal‐temporal area is interpreted as reduced empathy for the victim so that appetitive violence could occur (Moran et al., 2014). More specific neurobiological correlates of appetitively motivated perception of violence in subjects from the community have not been described so far although this form of violence is omnipresent in the media and the real world (e.g., hooliganism) (Elbert et al., 2010).

### 1.5 Hypotheses of our investigation

When individuals receive a reward, dopamine is released in the mesolimbic reward system (Urban et al., 2012). Individuals will be motivated by an increased dopamine transmission to act in a way perceived as rewarding (Bromberg‐Martin, Matsumoto, & Hikosaka, 2010). The mesolimbic dopaminergic reward system might be activated during perception and performance of appetitive violence (Kareken, 2018). We therefore assume that aggressive individuals who voluntarily and actively perform violent actions (martial artists) show a decreased activation in reward‐associated, cortical‐frontal, inhibitory areas (orbitofrontal cortex) and an increased activation in reward‐associated, subcortical areas (i.e., ventral striatum, especially nucleus accumbens as the target area of the reward system) and brain regions that are closely related to them (amygdala) (O'Doherty, 2004). A decreased frontal top‐down‐control may favor the disinhibition of emotion‐related subcortical areas (Potegal, 2012) and therefore elicit appetitive aggression (Elbert et al., 2010). Martial artists were selected for this study because we assumed that they possibly have a predisposition for performing and perceiving violence in a pleasurable way (Vertonghen, Theeboom, & Pieter, 2014). They might search for situations where they can act violently in a socially accepted form. The aim of this study was to investigate whether watching violence in contrast to neutral pictures leads to differentiated neuronal activation patterns of the nucleus accumbens, amygdala, and orbitofrontal cortex. The hypotheses underlying this study are that martial artists show higher activation of the nucleus accumbens and amygdala and less activation of the orbitofrontal cortex in contrast to controls. In more aggressive subjects, we expected a higher activation of the nucleus accumbens and amygdala and a reduced activation of the orbitofrontal cortex.

## 2 MATERIALS AND METHODS

### 2.1 Participants

*N* = 22 healthy male martial artists from local fight clubs and *n* = 26 healthy controls from the community were recruited. The size of our sample was reduced to *n* = 16 martial artists and *n* = 24 controls because of technical problems during functional magnetic resonance imaging (fMRI) data acquisition for *n* = 6 martial artists and because of the left‐handedness of *n* = 2 controls. All participants were right‐handed. The martial artists have practiced martial arts in different unarmed disciplines (*n* = 4 Muay Thai, *n* = 4 judo, *n* = 2 mixed martial arts, *n* = 2 kung fu, *n* = 1 boxing, *n* = 1 pankration, *n* = 1 Jiu‐Jitsu, *n* = 1 karate). They reported a mean experience of 8.36 ± 5.34 years in practicing martial arts while none of the controls had been trained martial arts at any time. All subjects gave their written informed consent according to procedures approved by the ethics committee of the Faculty of Medicine (Otto‐von‐Guericke University Magdeburg) prior to study inclusion.

### 2.2 Measures

The suitability of MRI was inquired using a questionnaire to avoid inclusion of subjects with contraindications (e.g., metallic objects in the body, tattoos, vessel operations). Aggressiveness was recorded using the FAF (aggressiveness factors questionnaire; Hampel & Selg, 1998). For further analyses the raw score on the FAF scale sum of aggression indicators (Σaggression) was used. The trait reflected by this value represents an externalized aggression potential. As a measure of verbal intelligence, the MWT‐B (version B of the multiple‐choice vocabulary intelligence test; Lehrl, 1999) was performed. To examine potential differences regarding aggressiveness and intelligence in both groups (martial artists vs. controls) we applied the Mann–Whitney‐*U*‐Test respectively.

### 2.3 Paradigm

Every trial started with presenting a fixation cross for a period of between 1 and 7 seconds (randomly jittered) before presenting a picture with either a violent (e.g., two people beating each other) or a neutral social interaction (e.g., people sitting in a café) for 1.25 s. Then the contour of a square or circle was projected on the picture for 0.50 s. The subject had to indicate which shape had been shown by pressing a button. After the response respectively after the disappearance of the contour, the picture was presented alone for 1.25 s. Then in 50% of the trials (at random) a reward followed, independently of the subjects’ response. Reward was indicated by the display of “25 Cent” on the screen. In case of no reward “0 Cent” was displayed.

The subjects were informed by the instruction that the reward would follow independently of their answer. To assure that the methods are sensitive enough to demonstrate activation of the brain reward system, a stimulus well known for its rewarding effect (money) was shown (25 Cent) in addition to pictures with violent scenes. The rewarding (hedonistic) aspect of appetitive aggression is measured while watching the violent pictures. To avoid that cognitive processing confounds the measurement of the appetitive aspect of aggression (e.g., subjects assess that violence is morally reprehensible) the cognitive cover task has been applied.

**Figure 1:**
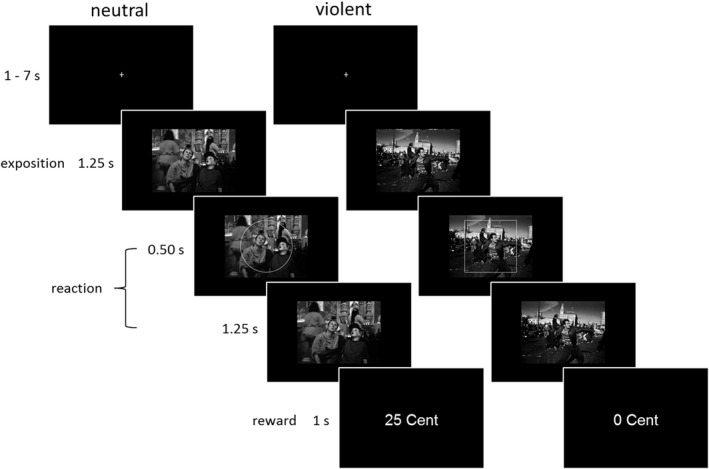
Two trials of the paradigm (s = seconds). On the left, a neutral trial is illustrated and one the right a violent one. After presenting a fixation cross, a neutral or violent picture follows. Then the contour of a square or circle is projected on the picture. The subject decides which shape is shown by pressing a button. Then a reward follows (i.e., 25 Cent appears on the screen) or not (0 Cent) at random, independently of the subjects’ response

One trial lasted at most 10 s. One block contained 36 trials. Eighteen violent and 18 neutral pictures were randomly presented in one block. One block lasted at most 6 min. The subjects had to perform three blocks. There was a short break after each block for 30–60 s. Figure 1 illustrates one neutral trial (left) and one violent trial (right).

### 2.4 MRI data acquisition

Structural images were obtained using a 3 Tesla Siemens (MAGNETOM Trio, Syngo MR A35; Siemens, Erlangen, Germany) MRI scanner with an eight‐channel phased‐array head coil. All subjects were given earplugs for noise protection in the head coil. Whole‐brain, T1‐weighted, 3D anatomical (MPRAGE, TR = 1,650 ms, TE = 5.01 ms, TI = 1,100 ms, FOV = 256 × 256 mm^2^, flip angle = 7 degree, 96 sagittal slices with a voxel size of 1.0 × 1.0 × 2.0 mm^3^ were obtained. Scan time for structural acquisition was 205 s. Functional images were acquired in three runs, each run lasted for 384 s and 190 volumes were acquired per run. The acquisition parameters were: 32 slices aligned to the AC‐PC line, slice thickness: 3.3 mm, 20% gap, TR = 2,000 ms, TE = 30 ms, flip angle = 80 degree, FOV = 208 × 208 mm^2^, voxel size: 3.3 × 3.3 × 3.3 mm^3^.

### 2.5 Analysis of the fMRI‐data

We used MATLAB R2015b and SPM12 (http://www.fil.ion.ucl.ac.uk/spm/) for data analysis. First, the data were preprocessed performing realignment, slice timing correction, normalization, and smoothing. Motion correction was done by rigid‐body realignment. Slice order was interleaved collecting first the even numbered slices followed by the odd‐numbered slices. Normalization was done to transform the brain images from each subject to reduce the variability between the subjects to allow meaningful group analyses. For this, the normalization procedure was performed by using MNI templates provided by SPM.

Then a first‐level general linear model (GLM) analysis was run on the whole brain using violent pictures, neutral pictures, reward (25 Cent), and nonreward (0 Cent) as regressors of interest, a 128 s high‐pass filter and a canonical hemodynamic response function (HRF). That is, the BOLD response of the stimulus data was modeled by convolving the HRF. Then the contrast values were computed for violent > neutral pictures. Using the Hammer atlas (Gousias et al., 2008; Hammers et al., 2003), we identified six regions of interest (ROI) defined by the shape of the anatomical structure: the left nucleus accumbens (with the coordinates *x* = −8.08, *y* = 8.38, and *z* = −9.01, 151 voxels), the right nucleus accumbens (*x* = 9.38, *y* = 9.29, *z* = −8.42, 139 voxels), the left amygdala (*x* = −22.31, *y* = −5.02, *z* = −20.50, 708 voxels), the right amygdala (*x* = 23.35, *y* = −3.54, *z* = −20.52, 764 voxels), the left orbitofrontal cortex (*x* = −23.28, *y* = 36.46, *z* = −16.76, 8,628 voxels), and the right orbitofrontal cortex (*x* = 24.66, *y* = 38.42, *z* = −16.46, 9,469 voxels). Thus, the ROIs were downsampled to match the fMRI resolution.

We also computed the contrast values for reward > nonreward as quality check to ensure that the reward system is activated. For this computation we used the same six regions of interest (bilateral nucleus accumbens, amygdala, orbitofrontal cortex). Average contrast values were then extracted from the six ROIs and entered in SPSS statistics 24 (IBM corp). These values were entered in six GLMs followed by *F* tests to investigate main effects and interactions of the group factor and FAF scores, using age as a covariate. We then applied FDR (false discovery rate; http://www.sdmproject.com/utilities/?show=FDR) as correction for multiple comparisons. The significance level was set to 95% (*α* = 0.05). Afterwards, the data were inspected to assess the directionality of the effect.

The structural images (T1) of all subjects were inspected to exclude brain tissue lesions because head injuries could function as confounder distorting the results (MinJoon, 2016).

## 3 RESULTS

The groups were aged‐matched (Table 1; *t* test for independent samples, *t(38)* = −0.39*, p* = 0.70; martial artists: 24.75 ± 4.04 years, controls: 25.42 ± 6.01 years). Both groups did not differ in their verbal IQ (Table 1; MWT‐B; Mann–Whitney‐*U*‐Test, *Z *= −1.79*, p* = 0.07), that was in the average range (martial artists: 108.50 ± 11.24, *Mdn* = 105.50, controls: 102.88 ± 10.61, *Mdn* = 100.05). Both groups differed significantly in their aggressiveness (FAF scale “∑aggressiveness”) with the martial artists being more aggressive (Table 1; Mann–Whitney‐*U*‐Test, *Z *= −1.97, *p* = 0.05; martial artists: *Mdn* = 12.00, controls: *Mdn* = 9.50).

**Table 1:** Sample characteristics (*M* = mean, *SD* = standard deviation, *Mdn* = median, p = significance level, IQ = intelligence quotient, MWT‐B = version B of the multiple‐choice vocabulary intelligence test, ∑=sum, FAF = aggressiveness factors questionnaire)

	Martial artists (*n* = 16)	Controls (*n* = 24)	Statistics
Age	*M* = 24.75	*M* = 25.42	*t(38)* = −0.39
	*SD* = 4.04	*SD* = 6.01	*p* = 0.70
IQ (MWT‐B)	*M* = 108.50	*M* = 102.88	*t(38)* = *1.60*
	*SD* = 11.24	*SD* = 10.61	*p* = 0.12
∑Aggressiveness (FAF)	*Mdn* = 12.00	*Mdn* = 9.50	*Z* = −1.97
			*p* = 0.05

In all subjects, no indications for brain tissue lesions were detected.

Across all participants, the right nucleus accumbens was activated when getting reward (25 Cent) in contrast to nonreward (0 Cent) (one sample *t* test, *t(39) = 2.65, p* < 0.05). The other regions of interest were not activated in this condition, each also tested by one‐sample *t* test: left nucleus accumbens, *t(38) = *0.81, *p* = 0.42; left amygdala, *t(39) = *1.19, *p* = 0.24; right amygdala, *t(39) = *1.16, *p* = 0.25; left orbitofrontal cortex, *t(39) = *1.60, *p* = 0.12; right orbitofrontal cortex, *t(39) = *0.74, *p* = 0.46.

**Figure 2:**
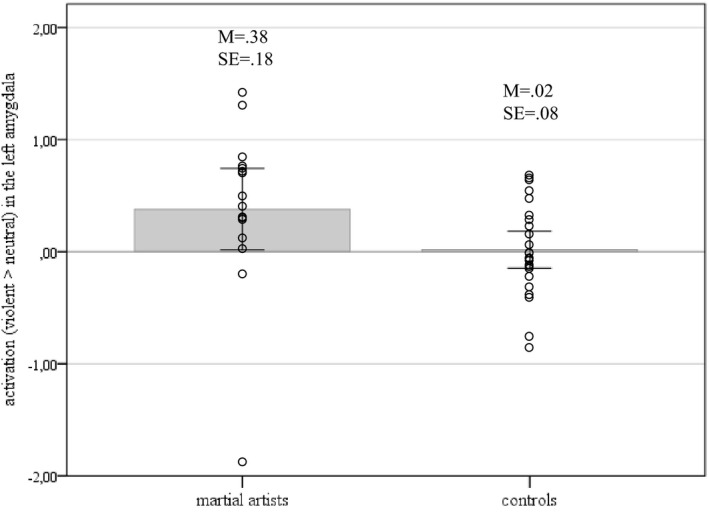
Martial artists in contrast to controls had a higher activation in the left amygdala when watching violent pictures (*M* = mean, SE = standard error)

On average, martial artists in contrast to controls had a higher activation in the left amygdala when watching violent pictures (*F_df = 1_*=12.59, *p* < 0.01). Figure 2 shows this main effect of the group.

**Figure 3:**
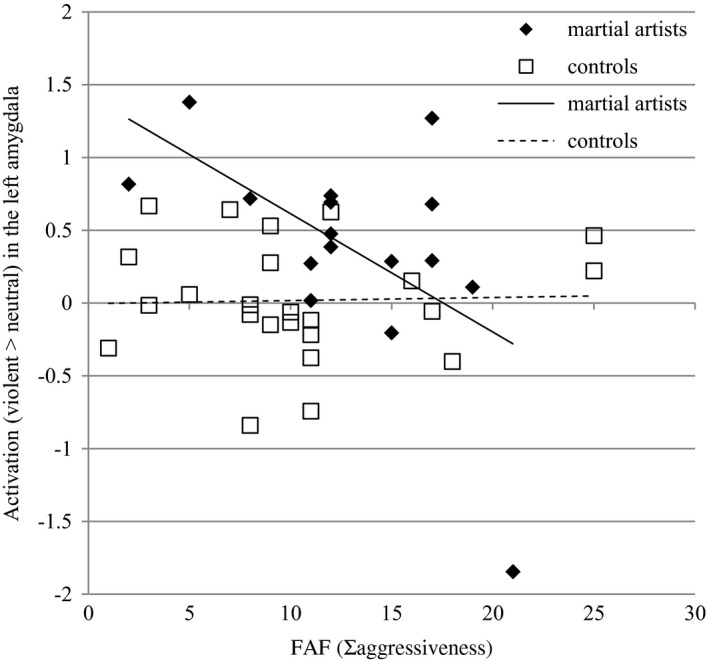
The more aggressive martial artists were (*x*‐axis), the smaller their activation in the left amygdala (*y*‐axis)

Furthermore, we found a group by FAF interaction effect (Figure 3). The more aggressive martial artists were, the smaller their activation in the left amygdala in this condition (*F_df = 1_*=7.66, *p* = 0.05). The relationship between FAF scores and amygdala reactivity is present in martial artists but not in controls.

No other main effect of the group was detected: The left nucleus accumbens (*F_df = 1_*=0.16, *p* = 0.92), the right nucleus accumbens (*F_df = 1_*=0.09, *p* = 0.92), the left orbitofrontal cortex (*F_df = 1_*=0.36, *p* = 0.92), the right orbitofrontal cortex (*F_df = 1_*=0.00, *p* = 0.97), and the right amygdala (*F_df = 1_*=1.82, *p* = 0.56) did not show a significantly changed activation when watching violent in contrast to neutral pictures. Furthermore, no main effect of the FAF scores was detected (left nucleus accumbens: *F_df = 1_*=0.00, *p* = 0.95; right nucleus accumbens: *F_df = 1_*=0.01, *p* = 0.95; left orbitofrontal cortex: *F_df = 1_*=4.61, *p* = 0.11; right orbitofrontal cortex *F_df = 1_*=3.90, *p* = 0.11; left amygdala: *F_df = 1_*=5.07, *p* = 0.11; right amygdala: *F_df = 1_*=0.11, *p* = 0.95). A group by FAF interaction effect in another ROI was not detected (left nucleus accumbens: *F_df = 1_*=0.41, *p* = 0.79; right nucleus accumbens: *F_df = 1_*=0.62, *p* = 79; left orbitofrontal cortex: *F_df = 1_*=0.03, *p* = 0.88; right orbitofrontal cortex: *F_df = 1_*=0.03, *p* = 0.88; right amygdala: *F_df = 1_*=0.46, *p* = 79). Table 2 summarizes the statistical results.

**Table 2:** Statistical results for the contrast violent versus neutral pictures for each ROI (region of interest; NAcc = nucleus accumbens, AMY = amygdala, OFC = orbitofrontal cortex) for the main effect of the group, the main effect of the FAF (aggressiveness factors questionnaire) and the interaction effect of group and FAF

ROI	Effect	*F*	*p*
Left NAcc	Group	0.16	0.92
	FAF	0.00	0.95
	Group and FAF	0.41	0.79
Right NAcc	Group	0.09	0.92
	FAF	0.01	0.95
	Group and FAF	0.62	0.79
Left AMY	Group	12.59	**<**0.01**
	FAF	5.07	0.11
	Group and FAF	7.66	0.05*
Right AMY	Group	1.82	0.56
	FAF	0.11	0.95
	Group and FAF	0.46	0.79
Left OFC	Group	0.36	0.92
	FAF	4.61	0.11
	Group and FAF	0.03	0.88
Right OFC	Group	0.00	0.97
	FAF	3.90	0.11
	Group and FAF	0.03	0.88

Note

*F* = empirical *F*‐value, *p* = significance level (set to *α* = 0.05).

* indicates *p* ≤ 0.05,

** indicates *p* ≤ 0.01.

## 4 DISCUSSION

This study was conducted in order to test the hypothesis that reward related brain structures are activated during perception of violent acts in individuals predisposed to appetitive performance and perception of violence. While, as predicted, the monetary reward paradigm of this study activated a core structure of cerebral reward systems (nucleus accumbens), this was not the case in martial artists by viewing violent pictures. Instead, martial artists (but not controls) had a higher activation in the left amygdala when watching violent pictures. The amygdala is known to be activated by affectively and motivationally salient stimuli (Rosell & Siever, 2015), for example, watching people beating each other. Furthermore, the activation of the amygdala is, at least in primates, known to increase when the reward increases what may also reflect increased salience (Bermudez & Schultz, 2010). In humans, the left amygdala is involved in explicit/conscious mechanisms of affect processing whereas the right amygdala is connected to more implicit/automatic mechanisms of affect processing (Rosell & Siever, 2015). Enhanced amygdala activation has previously been observed in individuals with intermittent explosive disorder after they had been presented with angry or fearful faces (Coccaro, McCloskey, Fitzgerald, & Phan, 2007; McCloskey et al., 2016). This finding is well in line with the notion of increased salience of the faces to these individuals. The violent pictures in our study also depict angry (offender) and fearful (victim) faces. The failure to resist impulses is the cardinal symptom of intermittent explosive disorder (DSM‐5, APA, 2013) which often leads to impulsive aggressive behavior (Look, McCloskey, & Coccaro, 2015). In the present study martial artists were more aggressive as compared to controls (as assessed by FAF, martial artists reported a higher degree of externalized aggression). They exerted this specific hobby probably because they may enjoy acting violently in a socially accepted manner. Supporting this assumption, persons with more physical aggression and conduct problems appeared to be attracted by more violent combat sports (Vertonghen et al., 2014). In a competitive reaction time task (Buades‐Rotger & Krämer, 2018), a positive relationship between the attention to antisocial cues and aggressive behavior was found exclusively in subjects that showed enhanced amygdala activation when viewing angry faces. It should be noted that in this study the subjects were female and that words instead of pictures were used as cues. Nonetheless the prolonged attention to antisocial cues that was observed in this study might reflect that angry face cues conveyed increased salience to these subjects. Accordingly, in our study the violent pictures might also convey a stronger salience to the martial artists than to the controls. On the neuronal level this might be reflected by an increased neuronal activation of the amygdala (Morrison & Salzman, 2010; Murray, 2007). Furthermore, higher level of affective arousal is known to be reflected by stronger amygdala activation (Touroutoglou, Bickart, Feldmann Barrett, & Dickerson, 2014).

Within the group of martial artists the extent of aggressiveness was inversely correlated with activation in the left amygdala when watching violent pictures. At first glance this seems contradictory to the stronger amygdala activation in martial artists as compared to the control group. One explanation is that more aggressive martial artists might show better adaptation to aggressive situations because they participate in training sessions or competitions more often than the less aggressive martial artists. Such a habituation might result in lower amygdala activation (Plichta et al., 2014). In a study applying a frustration task (Pawliczek et al., 2013), highly aggressive subjects showed a decreased activation in the left amygdala, too.

Functional deficits in the orbitofrontal cortex have been frequently described in psychopaths (e.g., Anderson & Kiehl, 2014) but not in subjects from the community like our sample. Buckholtz et al. (2010) found a positive relationship between the activation in the left nucleus accumbens and impulsive‐antisocial traits in a sample from the community when performing a “Monetary incentive delay task”. This correlation referred to monetary reward anticipation and not to the rewarded perception of violence. Methodical limitations could be a possible explanation why we did not find reward‐associated activation changes in the orbitofrontal cortex and nucleus accumbens during violence perception.

### 4.1 Limitations

The sample size (*n* = 16 martial artists, *n* = 24 controls) is relatively small. Moreover, the experimental group practiced different martial arts disciplines. In general, there are “softer” martial arts (like aikido) and “harder” combat sports (like kickboxing) (Vertonghen et al., 2014). Combat sports are characterized by full‐contact and a competitive aspect. The type of martial art respectively combat sport refers to different techniques that are predominantly applied; for example, karate and Muay Thai are striking‐predominant sports, judo and Jiu‐Jitsu are submission‐predominant sports (Jensen, Maciel, Petrigliano, Rodriguez, & Brooks, 2016) and mixed martial arts athletes combine the techniques of grappling and striking (James, Haff, Kelly, & Beckman, 2017). A stricter distinction of different martial arts should be taken into account in future studies. Furthermore, martial artists may differ in levels of impulse control associated with their chosen type of martial art, for example, mixed martial arts practitioners are characterized as more impulsive than boxers (Banks et al., 2014). Kick‐/Thaiboxers differ in physical aggression from athletes practicing judo, aikido, or karate (Vertonghen et al., 2014). People with a predisposition for such behavior may be attracted to these “harder” martial arts disciplines. Athletes may differ in the underlying motivation to practice martial art. Besides the possibility to act out aggressive impulses, this may be the intention to acquire self‐defense techniques or to improve physical fitness (Burke, Protopapas, Bonato, Burke, & Landrum, 2011; Vertonghen et al., 2014). Thus, appetitive components might not be the only motivation to practice martial arts. We did not use a psychometric rating instrument for the subjective pleasure levels of the subjects when watching violent pictures, since we assumed that the population practicing martial arts in contrast to controls practicing no martial arts might in general experience more pleasure while exerting and looking at violent scenes. Yoder, Porges, and Decety (2015) reported a positive relationship between watching and liking MMA scenes as well as watching and participating in MMA.

We assessed the appetitive aspect of aggression when subjects watched violent pictures while performing a cognitive task (pressing a button dependent on a square or circle that was depicted on the picture). It is conceivable that a stronger neuronal response in reward‐associated areas may be elicited by violent pictures without a cover task.

### 4.2 Outlook

The appetitive aspect of aggression is a condition of violence widely neglected so far despite it may be a universal trait detectable in many people (Moran et al., 2014). Following this assumption, appetitive aggression could be regarded as a proximate factor (according to the GAM) located in the person as a trait. The GAM does not contain neurobiological modifiers. Therefore we think that further neurobiological research about the appetitive aspect of aggression that addresses the reward system and that can be defined as a distal factor according to the GAM, is needed.

Neurobiological aspects including mechanisms of appetitive aggression should also be regarded as an essential component of violence.

## CONFLICT OF INTEREST

The authors declare that they have no conflict of interest.
